# Accessing Position Space Wave Functions in Band Structure Calculations of Periodic Systems—A Generalized, Adapted Numerov Implementation for One-, Two-, and Three-Dimensional Quantum Problems

**DOI:** 10.1021/acs.jpclett.3c01707

**Published:** 2023-08-11

**Authors:** Jakob Gamper, Florian Kluibenschedl, Alexander K. H. Weiss, Thomas S. Hofer

**Affiliations:** † University of Innsbruck, Theoretical Chemistry Division Institute of General Inorganic and Theoretical Chemistry, Center for Chemistry and Biomedicine, Innrain 80-82, A-6020 Innsbruck, Austria; ‡ Institute of Science and Technology Austria (ISTA), Am Campus 1, 3400 Klosterneuburg, Austria; ¶ University of Innsbruck, Research Institute for Biomedical Aging Research, Rennweg 10, A-6020 Innsbruck, Austria

## Abstract

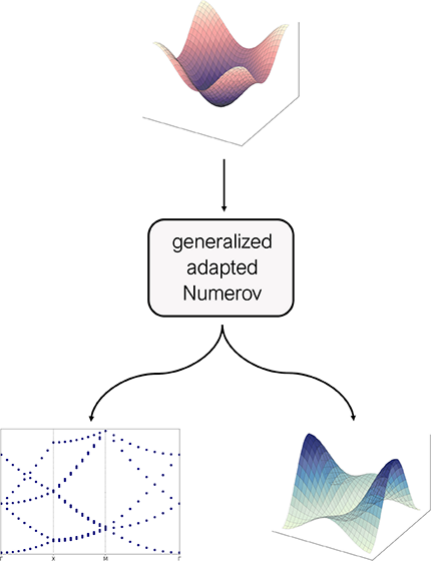

In this work, a generalized, adapted Numerov implementation capable of determining band structures of periodic quantum systems is outlined. Based on the input potential, the presented approach numerically solves the Schrödinger equation in position space at each momentum space point. Thus, in addition to the band structure, the method inherently provides information about the state functions and probability densities in position space at each momentum space point considered. The generalized, adapted Numerov framework provided reliable estimates for a variety of increasingly complex test suites in one, two, and three dimensions. The accuracy of the proposed methodology was benchmarked against results obtained for the analytically solvable Kronig-Penney model. Furthermore, the presented numerical solver was applied to a model potential representing a 2D optical lattice being a challenging application relevant, for example, in the field of quantum computing.

The Schrödinger picture is the fundamental mathematical description of quantum mechanics describing virtually every known physical and chemical phenomenon.^1^ In order to investigate the vibrational spectra of molecules, the coupling strength of different vibrations, or their respective spectral intensities, a number of different methods have emerged to numerically solve the nuclear Schrödinger equation, the most important being the grid-based Numerov method,^2,3^ discrete variable representation (DVR) techniques,^4,5^ the Chebychev collocation approach^6,7^ or vibrational SCF^8^ and vibrational perturbation theory.^9^ One of the most recent developments in this area is the adapted Numerov method, which implements an efficient and scalable approach to retrieving spectral information on a molecular basis.^10−12^ Recently, the quantum mechanical study of solid-state systems, including the determination of their associated band structures, has become increasingly important. The most prominent periodic model system for representing solid-state systems, the Kronig-Penney model, has been the subject of numerous scientific publications.^13−16^ The associated Schrödinger equation can be solved analytically, providing access to the corresponding dispersion relation in terms of the associated band structure. However, the Kronig-Penney model is only a very simple model system that does not represent the complexity of real solid-state systems. Therefore, the general investigation of more complex solid-state systems featuring more evolved potential energy surfaces requires numerical solutions to the Schrödinger equation. A prime example for such solid-state systems is so-called optical lattices, which play an important role in the advent of quantum computing. One way to enhance the physical properties and capabilities of quantum computers is to introduce and refine the applied optical lattices.^17−20^

In this Letter, a generalized approach of the adapted Numerov method is presented enabling the treatment of periodic solid-state systems for arbitrary momentum points in the Brillouin zone, thus allowing the calculation of the associated band structures. The previously introduced adapted Numerov approach^11,12^ is extended to also encompass the constraints imposed by the Bloch theorem,^21,22^ according to which the wave function is subject to a periodic potential. In this case, the wave function is given by
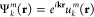
1with crystal momentum **k**, a scalar index k representing a unique identifier for **k**, band index m and the lattice periodic function *u*_*k*_^*m*^. To obtain *u*_*k*_^*m*^, the following equation has to be solved
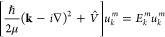
2

which is obtained by inserting the Bloch wave function *u*_*k*_^*m*^ into the time-independent Schrödinger equation, with V̂ being the potential energy operator of the system, *ℏ* the reduced Planck constant,^23^ μ the effective mass, and ∇ the Nabla operator. Unlike other strategies for determining the band structure of a system of interest, the approach presented here solves the generalized Schrödinger equation in position space. To obtain information about the full band structure, the equation must be solved at each point of interest in the momentum space.

The generalized Schrödinger equation can be applied in the most general form of the adapted Numerov method, which is given by
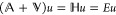
3

corresponding to a matrix eigenvalue equation. As in the original adapted Numerov method, no information about the wave functions has to be given; only the determination of the sparse matrix  differs from the original implementation.

To construct the matrix , the numerical difference matrix  of the adapted Numerov method has to be extended to the following form
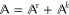
4where  represents the momentum-dependent part of the matrix.

Comparing the kinetic energy operator of the Schrödinger equation with the adapted Numerov method, the construction of the momentum space depends only on additive terms of the generalized Schrödinger equation
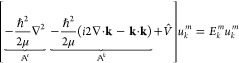
5

Thus, in order to construct the matrix formulation, the Laplacian of the generalized Schrödinger equation can be expressed via the same numerical differentiation scheme represented as a stencil matrix as proposed earlier.^11^ The momentum-dependent part of the matrix can be constructed by the square norm of the momentum vector represented as a diagonal matrix and the imaginary part depending on the Nabla operator expressed via finite differences analogous to the Laplacian. To construct the full moment-dependent matrix, the following matrix kernel can be derived for an exemplary 2D  matrix and an error of O(*h*^4^) for the central finite differences, thus using a 5-point stencil.
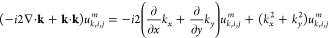
6
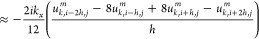
7
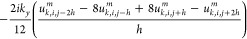
8
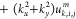
9

This leads to the following finite difference matrix kernel for the momentum-dependent part of the matrix
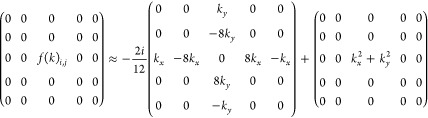
10

The construction of the 3D kernel matrix is implemented analogously to the 2D case by adding the corresponding finite difference matrix kernels for the *z*-direction. The resulting matrix is then added to the original adapted Numerov matrix . In order to outline the individual steps required to set up the Hamiltonian matrix , the workflow is described in greater detail in the Supporting Information at the example of a 5-point stencil implementation.

A noteworthy aspect of this extension is that the momentum-dependent matrix kernel features an even higher number of zero entries than the Laplacian kernel, and no elements of the momentum-dependent kernel overlap with zero entries of the Laplacian kernel, giving exactly the same degree of sparsity for the full matrix equation as for the original adapted Numerov framework.

As the presented work is based on periodic systems, the approach shows a different degree of sparsity compared to the adapted Numerov approach employing Dirichlet boundary conditions.^24^ The dimensions of the matrix eigenvalue equation can be constructed from the dimensions of the domain, on which the generalized Schrödinger equation is solved. Given a *D*-dimensional grid, the number *n*_*nz*_ of nonzero entries of the matrix can be estimated by the following equation
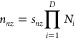
11

where *s*_*nz*_ represents the number of nonzero entries of the *s*-pointwise stencil kernel matrix and *N*_*i*_ the number of grid points in the *i*-th dimension. For the sake of generality, no closed formula for the number of nonzero entries in the kernel matrix is given, since the presented approach can be easily adapted in order to apply different finite differentiation schemes. However, in Table 1, the number of nonzero entries for the 1D, 2D, and 3D cases applying an 11-point stencil for a central differentiation scheme is given. Furthermore, also the degree of sparsity is included in the table, which is defined as the ratio of nonzero entries to the total number of entries in the matrix.

**Table 1:** Comparison of the Number of Nonzero Elements in the Hamiltonian Matrix for 1D, 2D, and 3D Potential Energy Grids with Different Grid Sizes, Ranging from 25 to 1000 along Each Dimension for an 11-Point Stencil

*D*	*N*	*N*^2*D*^	*s*_*nz*_	*n*_*nz*_	in %
1	25	625	11	275	44.0
1	50	2500	11	550	22.0
1	100	10000	11	1100	11.0
1	250	62500	11	2750	4.4
1	500	250000	11	5500	2.2
1	1000	1000000	11	11000	1.1
2	25	390625	85	53125	13.6
2	50	6250000	85	212500	3.4
2	100	100000000	85	850000	0.85
2	250	3906250000	85	5312500	0.136
2	500	62500000000	85	21250000	0.034
2	1000	1000000000000	85	85000000	0.0085
3	25	244140625	439	6859375	2.810
3	50	15625000000	439	54875000	0.351
3	100	1000000000000	439	439000000	0.0439
3	250	244140625000000	439	6859375000	0.00281
3	500	15625000000000000	439	54875000000	0.000351
3	1000	1000000000000000000	439	439000000000	0.0000439

The total number of nonzero elements *n*_*nz*_ is strongly dependent on the size *s* of the pointwise stencil employed to the generalized Numerov method; thus Tables S2 to S3 of the Supporting Information contain the same sparsity analysis for a 5-point, 7-point, and 9-point stencil implementation, respectively.

The presented generalized, adapted Numerov framework has been implemented in the programming language Julia.^25^ The matrix eigenvalue equation inherent to the generalized, adapted Numerov method can be solved via some basic algebraic manipulations, i.e., using a dense matrix algorithm from the LAPACK library^26^ for symmetric matrices. Considering the generalized, adapted Numerov approach, dense matrix algorithms have the disadvantage that the computational complexity increases exponentially with an increasing number of grid points, especially for problems of higher dimensionality. One possibility to tackle this obstacle is to use specially designed solvers to compute only a few eigenvalues and eigenvectors of the matrix eigenvalue problem. Within this work, a Krylov subspace projection method^27^ from the ARPACK library^28^ implemented for sparse matrices was used for all calculations. This not only reduces the computational complexity by restricting the computation to the lowest *n* eigenvalues and eigenvectors but also dramatically reduces the memory requirements and execution times of the algorithm by applying a sparse matrix solver.

The effective execution times for large problem sizes in higher dimensions for the sparse algorithms are orders of magnitude lower than those for the dense algorithms. However, it was found that the dense matrix algorithms performed better in terms of accuracy for larger matrix sizes without reconfiguring the settings of the applied algorithms for each specific calculation setup.

The presented generalized, adapted Numerov framework is introduced based on three exemplary systems of increasing complexity. On the one hand, the convergence of the approach is examined, and on the other hand, it is applied to the most prominent analytically solvable model system, the Kronig-Penney model. The performance with respect to 1D, 2D, and 3D variants of the model system is investigated. Furthermore, the method will be used to determine the band structure of an exemplary optical lattice potential, which represents a challenging real world application relevant to the development of quantum computers. These three test sets cover the most important aspects of the presented approach, namely convergence, accuracy, and applicability to real world problems. Where not explicitly stated, the calculations were performed using atomic units and 11-point stencils. Furthermore, the definitions for the most important momentum space points and their associated minimal k-path through the Brillouin zone are taken from ref (29).

In order to investigate the performance of the generalized, adapted Numerov approach for different momentum vector values and the resulting convergence with respect to the grid spacing, three band structure calculation scans were performed for a model cosine potential in 1D, 2D, and 3D. The cosine potential is given by
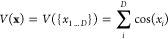
12where *D* is the dimensionality of the benchmark suit and *x*_*i*_ the position in the *i*-th dimension applied to a range from −π to π. For the 1D and 2D cases, 23 different grid spacings were employed, with a total of 12 to 100 grid points per domain. For the 3D case, 13 different grid spacings were used, with up to 64 grid points per domain. An 11-point stencil was used for all band structure calculations.

In order to analyze the grid spacing dependency of the convergence, the band energy values with the highest number of grid points were included as reference values. With these reference values, the relative logarithmic deviations of the band energy values were determined according to .

Figure 1 shows the results of the convergence analysis for all three-dimensionalities based on the ground state energy and the first two excitation energies at the most important points in the momentum space. For the one-dimensional approach, only the Γ and *X* points of the Brillouin zone are reported. In the case of the 2D approach the *M* and for the 3D approach the *M* and *R* points are also included.

**Figure 1:**
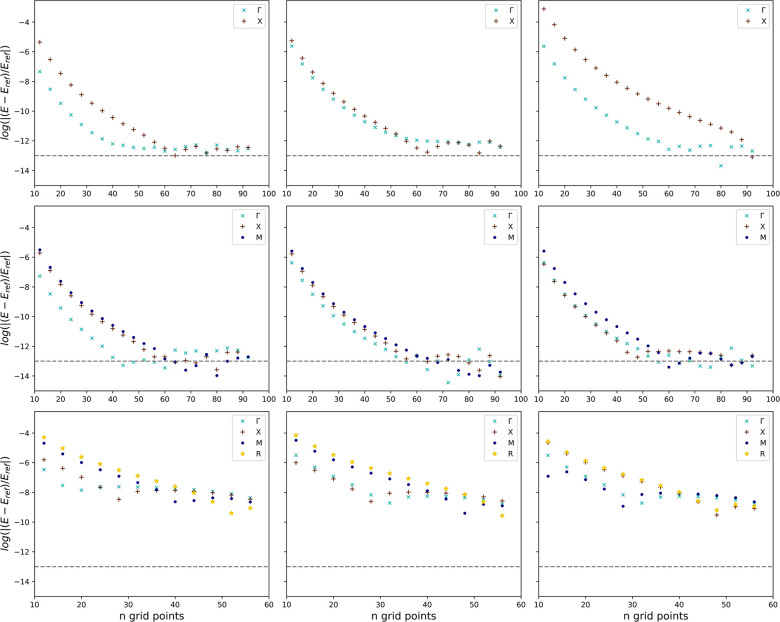
Graphical representation of the convergence of the generalized Numerov approach employed with an 11-point stencil for the sum of cosine potentials in 1D (top row), 2D (central row), and 3D (bottom row). The logarithmic relative deviation to a reference calculation, i.e., the calculation with the highest number of grid points (100 for the 1D and 2D potential and 64 for the 3D potential), is plotted against the number of grid points. The first column represents the convergence benchmark for the ground state energies, the second column for the first excited state energies, and the third column for the second excited state energies. The Γ point is represented by cyan markers, the *X* point by red markers, the *M* point by blue markers, and the *R* point by yellow markers.

Comparing the convergence of the different dimensionalities, it can be clearly stated that the higher the dimensionality of the approach, the lower the number of required grid points to achieve relative convergence. This holds true not only for all Brillouin zone points considered but also for higher excitations.

On the other hand, the 3D case shows a much lower level of convergence in terms of relative energy deviations compared to the 1D and 2D cases. The latter two reach the double precision limit of approximately 10^–13^ for both momentum space points and energy excitation levels.

Furthermore, for the dependency of the convergence on the momentum space points, it can be stated that the greater the distance in the reciprocal space from the center point of the domain, i.e., the Γ point, the greater the number of required grid points in order to achieve convergence.

In addition to the convergence benchmarks, the generalized, adapted Numerov method was tested against the analytically solvable Kronig-Penney model. Three calculations were performed in 1D, 2D, and 3D employing 51 grid points along each dimension ranging from −1.0 to 1.0 bohr. The potential was set to 0.0 hartree within the inner domain, i.e., the absolute value of the position has to be less than 0.5 bohr and 1.0 hartree in all dimensions in the outer domain. The potentials for the 1D and 2D cases are given in Figure 2a and Figure S1a in the Supporting Information, respectively. No visual representation is given for the 3D potential. The band structure of the 1D Kronig-Penney model is shown in Figure 2b represented by cross markers. In addition, the analytical band structures are listed as solid lines. From this band structure calculation, it can be seen that the newly introduced method perfectly reproduces the analytical reference data. Table S4 in the Supporting Information gives the absolute energy deviations for the eight lowest eigenvalues of three selected points in the momentum space. It can be seen that the deviations increase slightly for higher excitation energies with a maximum deviation of 1.4 × 10^–9^ hartree for the eighth eigenvalue. Furthermore, no significant difference in the performance can be observed for different state points at the same excitation levels.

**Figure 2:**
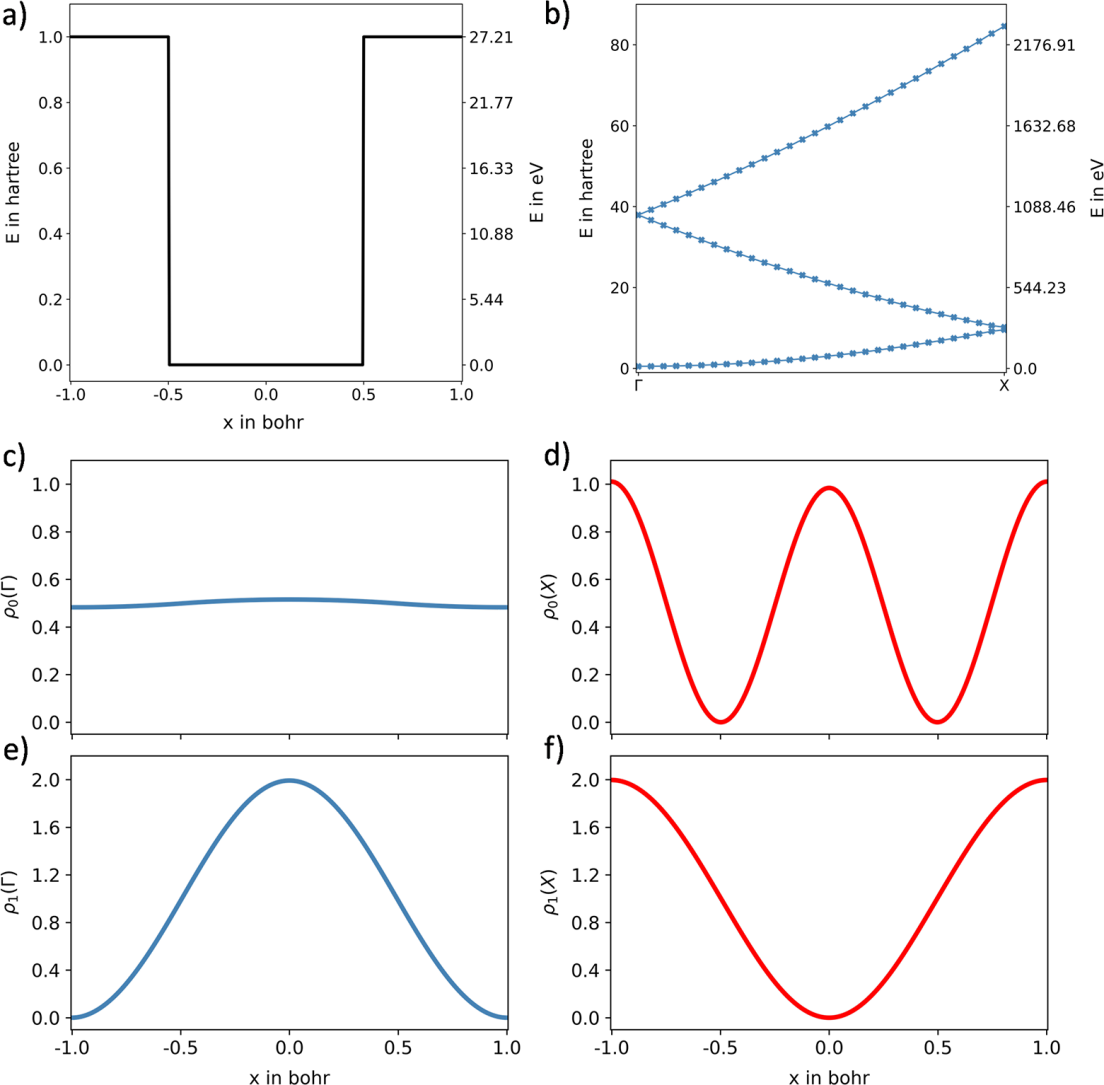
Visualization of the results for the 1D Kronig-Penney model obtained by applying the generalized, adapted Numerov method to a 51-point potential energy grid with an 11-point stencil. Depiction of (a) the periodic potential energy surface and (b) the associated band structure along the Γ-*X* path in momentum space. Probability densities ρ in position space for the ground state (central row) and first excited state (bottom row) at the Γ point are shown in parts (c) and (e), respectively. The probability densities for the *X* point are depicted analogously in (d) and (f).

The enormous advantage of solving the generalized Schrödinger equation in position space rather than in momentum space, as is done in the presented approach, is the possibility of solving the Schrödinger equation at any point in momentum space and inherently obtaining a numerical representation of the complex state function and its probability density in position space. Considering Figure 2c,d, the probability densities of the ground state at the Γ and *X* points are shown, respectively. Furthermore, Figure 2e,f represents the probability densities of the first excited state in an analogous manner.

The results for the 2D Kronig-Penney model can be found in the Supporting Information in Figure S1 with an equivalent figure setup as described above.

For the 3D Kronig-Penney potential, the resulting numerical band structure is shown in Figure 3a. The use of cross markers was again the preferred choice, since the solution of the adapted Numerov approach always yields the lowest *n* energy eigenvalues, while energy values belonging to the same band do not have to be associated with the same excitation level at the respective momentum space point. The isosurfaces of the probability densities for the ground state at the Γ, *X*, *M*, and *R* points are given in Figure 3b–e, respectively. For better visualizability, the isovalues of the subplots differ, due to the enormous change in the localization of the probability density at the different state points. While the probability density at the Γ and *R* positions is localized in the center of the cube, tunnel phenomena through the barrier of the Kronig-Penney potential can be observed along the *XY* plane for the *X* point and through the *YZ* plane for the *M* point (i.e., nonzero probability density).

**Figure 3:**
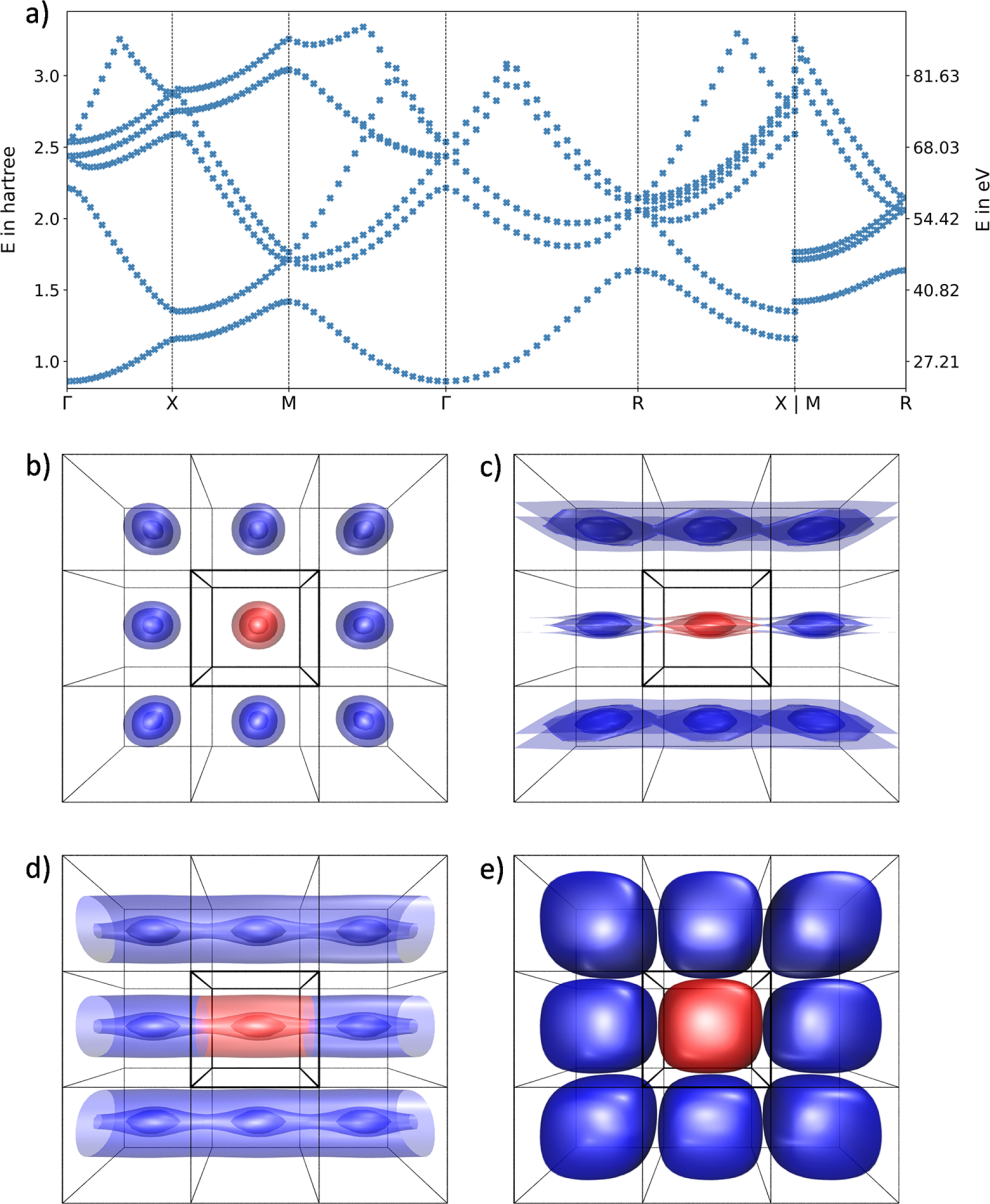
Graphical representation of the results for the 3D Kronig-Penney model calculation carried out using the generalized, adapted Numerov approach employed with an 11-point stencil and applied to a 51 × 51 × 51 potential energy grid. (a) Depiction of the band structure along the most important momentum space directions. (b–e) represent isosurfaces of a 3 × 3 × 1 unit cell of the ground state probability densities ρ_0_ in position space at the Γ, *X*, *M*, and *R* point, respectively.

In order to design suitable optical lattice potentials, e.g., for trapping and cooling atoms, the knowledge of the band structure is essential. Thus, the different excitation energies in the momentum space are of crucial importance. By applying the generalized, adapted Numerov method, the band structure of the 2D optical lattice potential can be calculated within a few seconds up to a few minutes depending on the number of grid points, lattice points, and excitation levels. Therefore, it is possible to perform thousands of calculations in a short period of time to construct the optical lattice potential that meets the desired requirements.

In the context of this work, an exemplary 2D optical lattice calculation was performed based on the following model potential in atomic units:
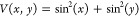
13

The described potential of the 2D optical lattice is shown in Figure 4a for 51 grid points along each dimension ranging from − bohr to  bohr. Furthermore, the band structure is given in Figure 4b, while Figure 4c,e,g represents the probability densities of the ground state at the Γ, *X*, and *M* points, respectively. The probability densities of the first excited state are given analogously in Figure 4d,f,h.

**Figure 4:**
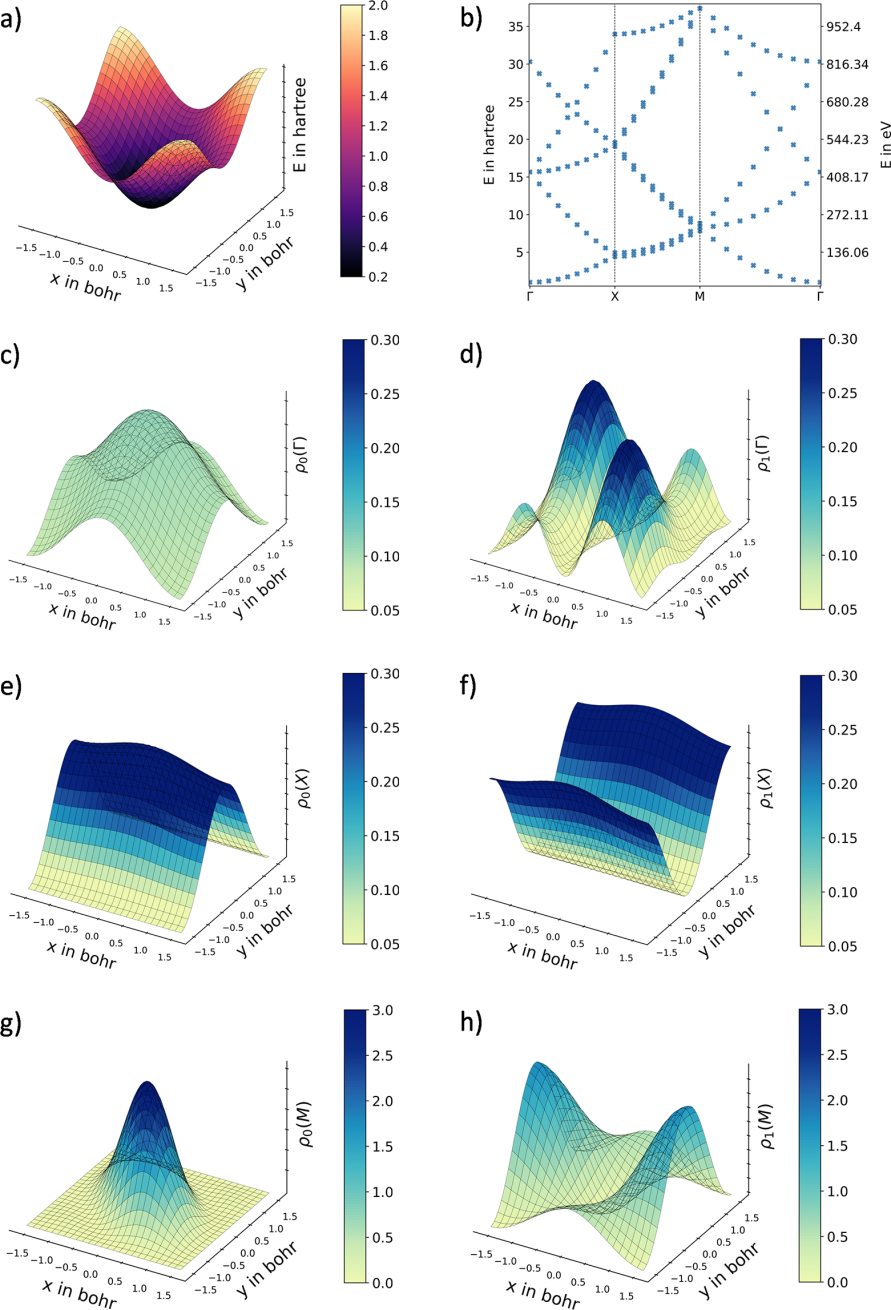
Visualization of the exemplary 2D optical lattice potential solved in the position space with the generalized, adapted Numerov approach employed with an 11-point stencil to a potential energy surface of 51 × 51 grid points. (a) Depiction of the potential energy surface and (b) associated band structure along the most important momentum space directions. (c, d) show the probability densities in the position space of the ground state and the first excited state for the Γ point, respectively. (e–h) include the probability densities for the *X* and *M* points in the momentum space in an analogous way.

For the color representation of the ground and first excited state at the *M* point, a different color scale had to be used due to the expected high localization in the center of the unit cell. For the ground state at the Γ and *X* points, a nonzero probability density can be observed for this exemplary potential, indicating a tunnel probability of the particle and thus a delocalization of the particle in the position space. In contrast, the probability density of the ground state at the *M* point is highly localized in the center of the potential, indicating a zero tunnel probability.

For the first excited state at the Γ and *M* points, one would expect a symmetric probability density in accordance with the given input potential. Due to a degeneracy of the energy eigenvalues at these state points, the resulting probability densities are a superposition of the symmetric and antisymmetric states.

In this paper, a generalization of the adapted Numerov approach is presented, which allows one to access information on momentum space through a series of calculations in position space, thus allowing also the determination of band structures. The newly developed approach shows all of the advantages of the original adapted Numerov method, such as the high accuracy and the high computational efficiency. The high degree of sparsity remains exactly the same as for the native adapted Numerov method, with drastic reduction of the memory requirements compared to the original Numerov method. Furthermore, the newly developed approach is not limited to one-dimensional problems but can also be applied to two- and three-dimensional problems, with a straightforward extension of the algorithm to even higher dimensions.

A key advantage of the presented method is the approach to solving the momentum-space-dependent Schrödinger equation in position space, which enables the calculation of the complex state functions and their probability densities in position space. Due to the nature of the Numerov approach, the calculation of the complex state functions and their probability densities is inherent to the method and thus does not require any additional computational effort without the use of any approximated basis sets.

The presented approach has been applied to the calculation of the band structure of the 1D, 2D, and 3D Kronig-Penney models as well as to a 2D optical lattice potential. The values obtained from the band structure calculations are in agreement with the expected results, thus demonstrating the validity of the presented approach. Furthermore, the probability densities of the ground and first excited states at different state points of the Kronig-Penney model and the optical lattice potential were calculated and visualized.

The very efficient computational demands of the generalized Numerov approach should also be highlighted. For the largest system, the 3D Kronig-Penney model, the calculation of the full band structure with 116 momentum space points and 10 excitation levels took only 5 h, when running in parallel on a six-core Intel Core i7–6800K CPU @ 3.40 GHz processor. Furthermore, the presented algorithm is embarrassingly parallelizable for a full band structure calculation, without any further changes to the implementation. Since the Schrödinger equation is solved in position space, the solutions at different state points in momentum space are independent of each other, thus allowing the computations to be distributed across different cores or even different machines.

Regarding the practical application of the generalized Numerov approach, two possible scenarios have to be considered. First, the determination of the input potential is done via potential energy scans based on QM or QM/MM methods, and second, the potential is modeled via a continuous function that best describes the physical properties of the system of interest. In the first case, the time required for the determination of the potential energy surface is the time limiting factor, while the time consumption of the band structure calculation is negligible. For the second case, the presented approach can be used to further refine the designed model potential, taking into account the different excitation energies at different momentum space points by applying the approach to a multitude of different modifications of the model potential.

## Supporting Information Available

Examplary 2D matrix representation of the Hamiltonian; Sparsity analysis of the Hamiltonian matrix; Eigenvalues of the 1D Kronig-Penney model; Visualization of potential, band energies, and probability distributions obtained for the 2D Kronig-Penney model (PDF)
