# Supplementary material for: Accessing Position Space Wave Functions in Band Structure Calculations of Periodic Systems—A Generalized, Adapted Numerov Implementation for One-, Two-, and Three-Dimensional Quantum Problems
Source: J Phys Chem Lett. 2023 Aug 11;14(33):7395–403. doi: 10.1021/acs.jpclett.3c01707 (PMC10461292; doi:10.1021/acs.jpclett.3c01707)
Supplement: Supplementary file 1 — jz3c01707_si_001.pdf [file jz3c01707_si_001.pdf]

# Accessing Position Space Wave Functions in Band Structure Calculations of Periodic Systems - a Generalized, Adapted Numerov Implementation for One-, Two- and Three-Dimensional Quantum Problems

Jakob Gamper,[†] Florian Kluibenschedl,[†,‡] Alexander K. H. Weiss,[¶] and Thomas S. Hofer[*,†]

†*University of Innsbruck, Theoretical Chemistry Division, Institute of General, Inorganic and Theoretical Chemistry, Center for Chemistry and Biomedicine, Innrain 80-82, A-6020 Innsbruck, Austria*

‡*Institute of Science and Technology Austria (ISTA), Am Campus 1, 3400 Klosterneuburg, Austria*

¶*University of Innsbruck, Research Institute for Biomedical Aging Research, Rennweg 10, A-6020 Innsbruck, Austria*

E-mail: t.hofer@uibk.ac.at

## Examplary 2D Matrix Representation of the Hamiltonian

To provide further details of the proposed extension to the generalized adapted Numerov framework, an examplary 2D implementation of the Hamiltonian based on a 5-point stencil is provided.

Considering a 2D input potential energy surface $V(x, y)$ with N×M grid points, the Hamiltonian has to be implement as a $(N \cdot M) \times (N \cdot M)$ matrix. In order to construct the full Hamiltonian matrix $\mathbb{H}$ the following sum has to be evaluated:

$$\mathbb{H} = \mathbb{A}^r + \mathbb{A}^k + \mathbb{V}, \tag{1}$$

where $\mathbb{A}^r$ and $\mathbb{A}^k$ are the matrices representing the kinetic energy operator in the $x$ and $y$ direction (see eq. (3) to (5) in the main article), respectively, and $\mathbb{V}$ is the diagonal matrix representing the potential energy surface. To improve readability, the scalar indices $i \in [1, M]$ and $j \in [1, N]$ are introduced.

The potential energy matrix $\mathbb{V}$ is constructed by building a diagonal matrix from the linearized potential energy grid points $V_{i,i}$:

$$\mathbb{V} = \begin{pmatrix} V_{1,1} & 0 & 0 & \cdots & 0 & 0 & 0 & \cdots & 0 \\ 0 & V_{2,1} & 0 & \cdots & 0 & 0 & 0 & \cdots & 0 \\ 0 & 0 & V_{3,1} & \cdots & 0 & 0 & 0 & \cdots & 0 \\ \vdots & \vdots & \vdots & \ddots & \vdots & \vdots & \vdots & \ddots & \vdots \\ 0 & 0 & 0 & \cdots & V_{N,1} & 0 & 0 & \cdots & 0 \\ 0 & 0 & 0 & \cdots & 0 & V_{1,2} & 0 & \cdots & 0 \\ 0 & 0 & 0 & \cdots & 0 & 0 & V_{2,2} & \cdots & 0 \\ \vdots & \vdots & \vdots & \ddots & \vdots & \vdots & \vdots & \ddots & \vdots \\ 0 & 0 & 0 & \cdots & 0 & 0 & 0 & \cdots & V_{N,M} \end{pmatrix} \tag{2}$$

For the kinetic energy matrices $\mathbb{A}^r$ the following matrix kernel $\mathbb{K}^r$ has to be constructed

when considering a 5-point stencil:

$$\mathbb{K}^r = \frac{1}{720h^2}\begin{pmatrix} 0 & 0 & -1 & 0 & 0 \\ 0 & 0 & 16 & 0 & 0 \\ -1 & 16 & -60 & 16 & -1 \\ 0 & 0 & 16 & 0 & 0 \\ 0 & 0 & -1 & 0 & 0 \end{pmatrix}, \tag{3}$$

with $h$ being the grid spacing. From this kernel the kinetic energy matrix $\mathbb{A}^r$ can be build as a block matrix of $M^2$ submatrices $a^r_{i,j}$ of size $N \times N$ in the following form:

$$\mathbb{A}^r = -\frac{\hbar^2}{2\mu}\begin{pmatrix} a^r_{1,1} & a^r_{1,2} & \cdots & a^r_{1,M} \\ a^r_{2,1} & a^r_{2,2} & \cdots & a^r_{2,M} \\ \vdots & \vdots & \ddots & \vdots \\ a^r_{M,1} & a^r_{M,2} & \cdots & a^r_{M,M} \end{pmatrix} \tag{4}$$

In order to construct the submatrices $a^r_{i,j}$, the rows of the kernel $\mathbb{K}^r$ are transformed into banded matrices applying the following rules, displaying only elements associated to the kernel $\mathbb{K}^r$ (*i.e.* all elements not explicitly shown are zero):

<u>**case** $i = j$</u>

$$a^r_{i,i} = \frac{1}{720h^2}\begin{pmatrix} -60 & 16 & -1 & & & & & & -1 & 16 \\ 16 & -60 & 16 & -1 & & & & & & -1 \\ -1 & 16 & -60 & 16 & -1 & & & & & \\ & -1 & 16 & -60 & 16 & -1 & & & & \\ & & \ddots & \ddots & \ddots & \ddots & \ddots & & & \\ & & & -1 & 16 & -60 & 16 & -1 & & \\ & & & & -1 & 16 & -60 & 16 & -1 & \\ -1 & & & & & -1 & 16 & -60 & 16 & \\ 16 & -1 & & & & & -1 & 16 & -60 & \end{pmatrix} \tag{5}$$

**case** $|i-j-N\cdot round(\frac{i-j}{N})|=1$

$$a^r_{i,i+1}=\frac{1}{720h^2}\begin{pmatrix}16&0&0&&&&&0&0\\0&16&0&0&&&&&0\\0&0&16&0&0&&&&\\&0&0&16&0&0&&&\\&&\ddots&\ddots&\ddots&\ddots&\ddots&&\\&&&0&0&16&0&0&\\&&&&0&0&16&0&0\\0&&&&&0&0&16&0\\0&0&&&&&0&0&16\end{pmatrix}\tag{6}$$

**case** $|i-j-N\cdot round(\frac{i-j}{N})|=2$

$$a^r_{i,i+2}=\frac{1}{720h^2}\begin{pmatrix}1&0&0&&&&&0&0\\0&1&0&0&&&&&0\\0&0&1&0&0&&&&\\&0&0&1&0&0&&&\\&&\ddots&\ddots&\ddots&\ddots&\ddots&&\\&&&0&0&1&0&0&\\&&&&0&0&1&0&0\\0&&&&&0&0&1&0\\0&0&&&&&0&0&1\end{pmatrix}\tag{7}$$

For any other case not stated above, all entries of the submatrices $a^r_{i,j}$ will become zero. The construction of the submatrices $a^r_{i,j}$ can also be done *via* the columns of the kernel matrix $\mathbb{K}^r$ due to its symmetric properties. Furthermore, the second and third case include explicit 0 entries, which could be omitted in this special case of the 5-point stencil. However,

for the sake of generality, the explicit 0 entries are kept in the respective submatrices.

Applying the rules established for the submatrices, it can be seen that also the full kinetic energy matrix $\mathbb{A}^r$ can be constructed as a banded matrix of the submatrices $a^r_{i,j}$ in the following way by considering only the three different submatrices $a^r_{i,i}$, $a^r_{i,i+1}$ and $a^r_{i,i+2}$:

$$\mathbb{A}^r = -\frac{\hbar^2}{2\mu} \begin{pmatrix} a^r_{i,i} & a^r_{i,i+1} & a^r_{i,i+2} & & & & & a^r_{i,i+2} & a^r_{i,i+1} \\ a^r_{i,i+1} & a^r_{i,i} & a^r_{i,i+1} & a^r_{i,i+2} & & & & & a^r_{i,i+2} \\ a^r_{i,i+2} & a^r_{i,i+1} & a^r_{i,i} & a^r_{i,i+1} & a^r_{i,i+2} & & & & \\ & a^r_{i,i+2} & a^r_{i,i+1} & a^r_{i,i} & a^r_{i,i+1} & a^r_{i,i+2} & & & \\ & & \ddots & \ddots & \ddots & \ddots & \ddots & & \\ & & & a^r_{i,i+2} & a^r_{i,i+1} & a^r_{i,i} & a^r_{i,i+1} & a^r_{i,i+2} & \\ & & & & a^r_{i,i+2} & a^r_{i,i+1} & a^r_{i,i} & a^r_{i,i+1} & a^r_{i,i+2} \\ a^r_{i,i+2} & & & & & a^r_{i,i+2} & a^r_{i,i+1} & a^r_{i,i} & a^r_{i,i+1} \\ a^r_{i,i+1} & a^r_{i,i+2} & & & & & a^r_{i,i+2} & a^r_{i,i+1} & a^r_{i,i} \end{pmatrix} \quad (8)$$

The kinetic energy matrix $\mathbb{A}^k$ is constructed in an analogous way by using the kernel $\mathbb{K}^k$, which is given in the main manuscript (see eq. (11)). After addition of the three presented matrices $\mathbb{A}^r$, $\mathbb{A}^k$ and $\mathbb{V}$, the full Hamiltonian matrix $\mathbb{H}$ is obtained. The state functions $u^m_k$ and eigenenergies $E^m_k$ correspond to the eigenvectors and eigenvalues of the sparse Hamiltonian matrix $\mathbb{H}$, which can be determined *via* matrix diagonalization using any kind of (numerical) solver.

In order to compute the bandstructure of such a system, a user-defined number of Hamiltonians have to be constructed along a selected path through the Brillouin zone. By calculating the eigenvalues of each of these Hamiltonians, the different energy levels (band energies) at the different momentum space points $\mathbf{k}$ are obtained. It can be noted that for all Hamiltonians only the momentum space kinetic energy matrix $\mathbb{A}^k$ has to be modified, since the other two matrix $\mathbb{A}^r$ and $\mathbb{V}$ are independent of the actual value in $\mathbf{k}$.

Table S.1: Comparison of the number of non-zero elements in the Hamiltonian matrix for 1D, 2D and 3D potential energy grids with different grid sizes, ranging from 25 to 1000 along each dimension for a 5-point stencil.

| D | N | $N^{2D}$ | $s_{nz}$ | $n_{nz}$ | $\frac{n_{nz}}{N^{2D}}$ in % |
|---|---|---|---|---|---|
| 1 | 25 | 625 | 5 | 125 | 20.0 |
| 1 | 50 | 2500 | 5 | 250 | 10.0 |
| 1 | 100 | 10000 | 5 | 500 | 5.0 |
| 1 | 250 | 62500 | 5 | 1250 | 2.0 |
| 1 | 500 | 250000 | 5 | 2500 | 1.0 |
| 1 | 1000 | 1000000 | 5 | 5000 | 0.5 |
| 2 | 25 | 390625 | 9 | 5625 | 1.44 |
| 2 | 50 | 6250000 | 9 | 22500 | 0.36 |
| 2 | 100 | 100000000 | 9 | 90000 | 0.09 |
| 2 | 250 | 3906250000 | 9 | 562500 | 0.0144 |
| 2 | 500 | 62500000000 | 9 | 2250000 | 0.0036 |
| 2 | 1000 | 1000000000000 | 9 | 9000000 | 0.0009 |
| 3 | 25 | 244140625 | 33 | 515625 | 0.211 |
| 3 | 50 | 15625000000 | 33 | 4125000 | 0.0264 |
| 3 | 100 | 1000000000000 | 33 | 33000000 | 0.0033 |
| 3 | 250 | 244140625000000 | 33 | 515625000 | 0.000211 |
| 3 | 500 | 15625000000000000 | 33 | 4125000000 | 2.64e-5 |
| 3 | 1000 | 1000000000000000000 | 33 | 33000000000 | 3.3e-6 |

Table S.2: Comparison of the number of non-zero elements in the Hamiltonian matrix for 1D, 2D and 3D potential energy grids with different grid sizes, ranging from 25 to 1000 along each dimension for an 7-point stencil.

| D | N | $N^{2D}$ | $s_{nz}$ | $n_{nz}$ | $\frac{n_{nz}}{N^{2D}}$ in % |
|---|---|---|---|---|---|
| 1 | 25 | 625 | 7 | 175 | 28.0 |
| 1 | 50 | 2500 | 7 | 350 | 14.0 |
| 1 | 100 | 10000 | 7 | 700 | 7.0 |
| 1 | 250 | 62500 | 7 | 1750 | 2.8 |
| 1 | 500 | 250000 | 7 | 3500 | 1.4 |
| 1 | 1000 | 1000000 | 7 | 7000 | 0.7 |
| 2 | 25 | 390625 | 29 | 18125 | 4.64 |
| 2 | 50 | 6250000 | 29 | 72500 | 1.16 |
| 2 | 100 | 100000000 | 29 | 290000 | 0.29 |
| 2 | 250 | 3906250000 | 29 | 1812500 | 0.0464 |
| 2 | 500 | 62500000000 | 29 | 7250000 | 0.0116 |
| 2 | 1000 | 1000000000000 | 29 | 29000000 | 0.0029 |
| 3 | 25 | 244140625 | 67 | 1046875 | 0.429 |
| 3 | 50 | 15625000000 | 67 | 8375000 | 0.0536 |
| 3 | 100 | 1000000000000 | 67 | 67000000 | 0.0067 |
| 3 | 250 | 244140625000000 | 67 | 1046875000 | 0.000429 |
| 3 | 500 | 15625000000000000 | 67 | 8375000000 | 5.36e-5 |
| 3 | 1000 | 1000000000000000000 | 67 | 67000000000 | 6.7e-6 |

Table S.3: Comparison of the number of non-zero elements in the Hamiltonian matrix for 1D, 2D and 3D potential energy grids with different grid sizes, ranging from 25 to 1000 along each dimension for a 9-point stencil.

| D | N | $N^{2D}$ | $s_{nz}$ | $n_{nz}$ | $\frac{n_{nz}}{N^{2D}}$ in % |
|---|---|---|---|---|---|
| 1 | 25 | 625 | 9 | 225 | 36.0 |
| 1 | 50 | 2500 | 9 | 450 | 18.0 |
| 1 | 100 | 10000 | 9 | 900 | 9.0 |
| 1 | 250 | 62500 | 9 | 2250 | 3.6 |
| 1 | 500 | 250000 | 9 | 4500 | 1.8 |
| 1 | 1000 | 1000000 | 9 | 9000 | 0.9 |
| 2 | 25 | 390625 | 49 | 30625 | 7.84 |
| 2 | 50 | 6250000 | 49 | 122500 | 1.96 |
| 2 | 100 | 100000000 | 49 | 490000 | 0.49 |
| 2 | 250 | 3906250000 | 49 | 3062500 | 0.0784 |
| 2 | 500 | 62500000000 | 49 | 12250000 | 0.0196 |
| 2 | 1000 | 1000000000000 | 49 | 49000000 | 0.0049 |
| 3 | 25 | 244140625 | 197 | 3078125 | 1.26 |
| 3 | 50 | 15625000000 | 197 | 24625000 | 0.158 |
| 3 | 100 | 1000000000000 | 197 | 197000000 | 0.0197 |
| 3 | 250 | 244140625000000 | 197 | 3078125000 | 0.00126 |
| 3 | 500 | 15625000000000000 | 197 | 24625000000 | 0.000158 |
| 3 | 1000 | 1000000000000000000 | 197 | 197000000000 | 1.97e-5 |

Table S.4: Absoulte energy differences between the analytical dispersion relation and the generalized adapted Numerov solutions of the 1D Kronig-Penney potential for the lowest 9 energy eigenvalues at the Γ, $X/2$ and $X$ momentum space points. The generalized adapted Numerov framework was applied on a 51 point potential energy surface with an 11-point stencil.

| ΔE in hartree | Γ (k=0.0 $Å^{-1}$) | $X/2$ (k ≈ 0.78 $Å^{-1}$) | $X$ (k ≈ 1.57 $Å^{-1}$) |
|---|---|---|---|
| $\|\Delta E_0\|$ | <1E-12 | <1E-12 | <1E-12 |
| $\|\Delta E_1\|$ | <1E-12 | <1E-12 | <1E-12 |
| $\|\Delta E_2\|$ | <1E-12 | <1E-12 | <1E-12 |
| $\|\Delta E_3\|$ | <1E-12 | <1E-12 | <1E-12 |
| $\|\Delta E_4\|$ | <1E-12 | <1E-12 | <1E-12 |
| $\|\Delta E_5\|$ | <1E-12 | 1.1E-12 | 1.3 E-12 |
| $\|\Delta E_6\|$ | 3.0E-12 | 2.8E-12 | 6.2E-12 |
| $\|\Delta E_7\|$ | 3.2E-11 | 4.0E-11 | 4.6E-10 |
| $\|\Delta E_8\|$ | 7.3E-11 | 2.1E-10 | 1.4E-9 |

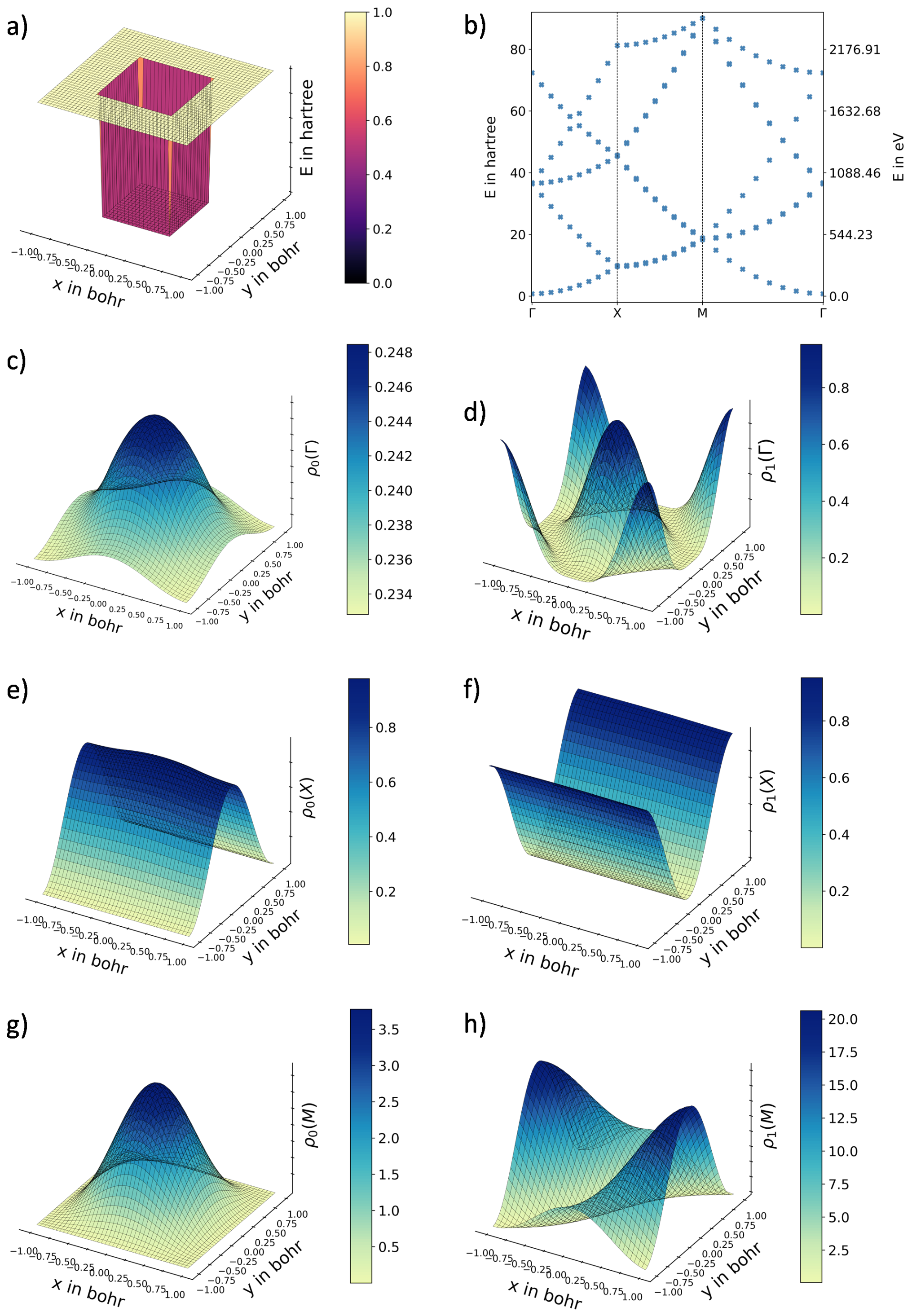


Figure S.1: Visualization of the 2D Kronig-Penney potential solved in the position space with the generalized adapted Numerov approach employed with an 11-point stencil to a potential energy surface of 51×51 grid points. a) Depiction of the potential energy surface and b) associated band structure along the most important momentum space directions. c) and d) show the probability densities in the position space of the ground state and the first exited state for the Γ point, respectively . e,f) and g,h) include the probability densities for the $X$ and $M$ points in the momentum space in an analogous way.
